# HIV and Neurocognitive Dysfunction

**DOI:** 10.1007/s11904-013-0171-y

**Published:** 2013-07-17

**Authors:** Serena Spudich

**Affiliations:** Department of Neurology, Yale University, 300 George Street, Room 8300c, New Haven, CT 06510 USA

**Keywords:** HIV/AIDS, HIV-associated neurocognitive disorder, Central nervous system, Antiretroviral therapy, Acute HIV infection, Primary HIV infection, Microglia, Cerebrospinal fluid, HIV-associated dementia, CNS reservoir, HIV reservoir, Compartmentalization, HIV escape, Viral load, Neurotoxicity, Efavirenz, Monocyte efficacy, Central nervous system penetration effectiveness, Blood–brain barrier

## Abstract

The spectrum of HIV-associated neurocognitive disorder (HAND) has been dramatically altered in the setting of widely available effective antiretroviral therapy (ART). Once culminating in dementia in many individuals infected with HIV, HAND now typically manifests as more subtle, though still morbid, forms of cognitive impairment in persons surviving long-term with treated HIV infection. Despite the substantial improvement in severity of this disorder, the fact that neurologic injury persists despite ART remains a challenge to the community of patients, providers and investigators aiming to optimize quality of life for those living with HIV. Cognitive dysfunction in treated HIV may reflect early irreversible CNS injury accrued before ART is typically initiated, ongoing low-level CNS infection and progressive injury in the setting of ART, or comborbidities including effects of treatment which may confound the beneficial reduction in viral replication and immune activation effected by ART.

## Introduction

More than 25 years after the initial recognition that HIV can directly damage the central nervous system (CNS) and lead to dementia [1], HIV-associated neurocognitive disorder (HAND) now encompasses a transformed spectrum of conditions due to the relative immune recovery or immune preservation facilitated by dramatic advances in antiretroviral therapy (ART). Though in the early days HAND presented as a severe motor and cognitive disorder in patients with advanced immunosuppression and AIDS, it now typically manifests as milder disturbances of psychomotor speed, processing speed, executive function, or memory [2••, 3]. Despite this much more subtle degree of impairment, HAND in the current era may affect daily function, antiretroviral adherence, and quality of life [4–6]. Severe forms of dementia, myelopathy, and neuropathy do still present in resource-limited settings, in patients with challenges to adherence, and in those who present late to HIV care. The fact that milder forms of HAND persist in resource rich settings despite the widespread use of effective ART [2••, 7, 8] remains a puzzle and challenge to providers and HIV-infected individuals. This review will focus on the current state of knowledge of the natural history of HIV in the CNS including establishment of CNS reservoirs for infection and injury, as well as address new issues and CNS disease entities emerging in the setting of antiretroviral therapy.

## Acute and Early HIV in the CNS and Effects of Early Treatment

Crucial processes in systemic HIV pathogenesis are initiated in acute and early HIV infection, including establishment of tissue HIV reservoirs, massive CD4 memory phenotype T cell loss, and establishment of viral and immune activation set points [9, 10]. The recognition that this period is crucial to disease pathogenesis and the possibility that early intervention with ART might preserve immune status and reduce seeding of viral reservoirs [11] has likewise raised the question of whether the nervous system is importantly affected by HIV during early HIV or may be protected by early treatment.

### HIV Neuroinvasion and Establishment of CNS Infection

Until recently, an understanding of the processes occurring in the CNS in the earliest stages of HIV infection have been informed almost entirely by animal models which allow for interrogation of the CNS after a known timing of initial lentiviral infection. In an accelerated rhesus macaque model of neuro-AIDS [12], CNS SIV invasion occurs in the first two weeks of infection, with detectable SIV in both the cerebrospinal fluid (CSF) and brain [13, 14]. Acute SIV is associated with brain infiltration of inflammatory T-cells, cytokine production and microglial activation [15–17], and the degree of elevation of CSF markers of immune activation (rather than CSF viral load) during acute and early infection is predictive of subsequent CNS disease [18]. SIV replication has been demonstrated in macrophages and microglia during the very early stages of infection in this accelerated model [19]. While RNA production is halted by initiation of treatment, levels of SIV DNA appear to be unaffected by combination ART, and measures of CNS inflammation may remain elevated in the setting of treatment [20•]. These studies suggest that acute SIV sets the stage for immune activation and infection in the CNS, and that a reservoir of infection may be established early during the course of disease which persists despite therapy. However, the generalizability of findings in an accelerated model with immune-depleted animals inoculated with neurovirulent HIV strains to the natural history of HIV in humans is uncertain.

**Fig. 1:**
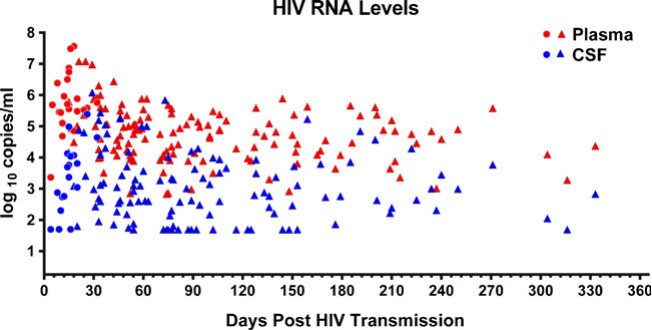
Paired HIV RNA levels in the blood (red) and cerebrospinal fluid (blue) compartments with respect to estimated days post HIV infection at initial sampling in antiretroviral naïve subjects with acute and primary HIV infection. Circles denote values measured in acute HIV subjects in Thailand with predominantly subtype CRF01_AE virus (adapted from [37], with permission); triangles denote values obtained in subjects from the USA, Sweden, Italy, and Australia with predominantly subtype B virus (updated from [38•], with permission). Note the approximately 15 percent of CSF samples with HIV RNA concentrations at or below the standard level of detection (50 copies/ml), often in the presence of high plasma HIV RNA, within the first 6 months after infection

In humans, acute HIV is associated with a systemic acute retroviral syndrome in up to 70 % of individuals [21]. In a subset of these, specific neurological signs and symptoms develop, including meningitis, encephalitis, mononeuropathies, brachial neuritis, meningoradiculitis, myelopathy and cauda equina syndrome [22–31]. The common features of these diverse disorders include emergence a few weeks after the onset of a systemic retroviral syndrome and a typically self-limited course, suggesting an autoimmune rather than HIV-specific etiology. However, in the majority of persons, HIV entry into the nervous system is neurologically asymptomatic, or associated only with headache [21, 32, 33]. Initial HIV neuroinvasion in humans has been known to occur within the first months of HIV, based on findings of HIV nucleic acid and proviral DNA within the cerebral cortex by day 15 after an iatrogenic acute HIV infection [34], and surveys of tissue compartments during the first months of HIV exposure [35, 36]. A recent systematic study of 96 subjects during primary HIV (within 12 months of infection) detected HIV in the CSF in a majority of individuals at a median 77 days post infection, indicating viral entry into the CNS compartment [37]. Of these subjects 15 % had CSF HIV RNA below the level of detection of standard assays in the setting of high plasma HIV RNA. In a separate study of subjects with acute HIV (median 14.5 days post infection) in Bangkok, Thailand, HIV RNA was measurable in CSF by a standard PCR assay in 15/18 subjects assessed within the first 30 days of estimated infection [38•], with higher median CSF HIV levels than in the primary infection cohort. This study also provided the earliest evidence of CNS entry of HIV in humans, measuring a viral load of 2.88 log_10_copies/ml in the CSF in one subject by 8 days post estimated infection. Taken together, data from these two cohorts suggest that HIV enters the CNS in the initial weeks after HIV exposure, but immune responses may control HIV RNA in the CSF in the early months of infection, in some individuals to undetectable levels (see Fig. 1). In chronic infection such local control is lost, with detectable CSF HIV a ubiquitous feature.

In chronic, established infection, HIV DNA has been recovered from homogenized brain tissue and specific cell types in the CNS including macrophages, microglia, and astrocytes, supporting the concept of anatomic and cellular reservoirs for HIV within the CNS [39–42]. Recent analysis of paired plasma and CSF samples from subjects with early HIV infection shows that in some cases early CSF HIV quasispecies are compartmentalized with respect to the blood, possibly suggesting an autonomous source of viral production in the CNS compartment within the first year after initial HIV transmission [43]. Overall, evidence of early seeding of the CNS compartment and examples of CNS compartmentalization of HIV species during early infection suggests that a CNS ‘reservoir’ for local HIV infection and production may need to be considered in treatment and HIV eradication strategies throughout the course of HIV infection.

### Pathogenesis of Acute and Early HIV in the CNS

Initial viral entry is fundamentally associated with evidence of CNS inflammation and immune activation, based both on CSF measures of cytokines and biomarkers of cellular activation and on neuroimaging measures which detect biochemical and cellular metabolites indicative of inflammation and gliosis within the brain parenchyma [37, 38•, 44, 45]. CNS immune activation is the putative substrate of CNS injury in HIV; neurons are not productively infected and neuronal dysfunction is thought to be mediated by indirect effects of macrophage and microglial activation and astrocyte dysfunction and possibly neurotoxic effects of viral proteins [46, 47]. Thus, an essential clinical question is whether presence of inflammation and HIV virions within the CNS might damage the brain during the early stages of infection, prior to typical diagnosis of HIV and initiation of treatment. Besides revealing prominent neuroinflammatory patterns in acute infection [38•, 45], neuroimaging measures have revealed mild reductions in brain imaging markers of neuronal integrity [48, 49] as well as evidence of brain atrophy [50], disruptions in brain connectivity networks [51], and reduced cerebral blood flow [52] in subjects assessed within the first year of infection. CSF markers may also be sensitive to subtle ‘subclinical’ CNS injury which may have longer-term consequences. Recently, mild elevations in CSF levels of the light subunit of neurofilament protein (NFL), indicative of active neuronal breakdown, were identified in almost half of a group of 92 largely neuroasymptomatic subjects at a median 3.1 months post infection [53•], consistent with a prior smaller report including some of the same subjects [54]. The strongest independent predictors of CSF NFL level in this group were CSF neopterin, a pteridine product produced by activated macrophages and microglia within the CNS, and CSF to plasma albumin ratio which rises with dysfunction of the blood brain barrier. CSF NFL levels also inversely correlated with brain neuroimaging markers of neuronal integrity. The clinical significance of the low level elevations in NFL seen in this group is uncertain, but these findings indicate that active injury to neurons does occur during even the early stages of HIV infection, and that this injury is closely associated with neuroinflammation and disruption of the blood brain barrier.

### Neurocognitive Function in Acute and Early Infection

Clinical studies have suggested that neuropsychological performance and indices of mood may be worse in subjects with early HIV infection compared with those in HIV-uninfected control subjects or published norms [55, 56]. Deficits are similar in pattern to those seen in subjects with chronic, treated HIV infection, including impairments in learning and processing speed. However, comorbidities may contribute to these differences, since subjects with recent HIV-infection by nature have higher rates of substance use, other sexually transmitted diseases, and mental health disorders than the general population. A recent examination of the contribution of co-morbid factors to these measures revealed that impaired neuropsychological performance correlated with methamphetamine use in subjects with recent HIV infection [57]. This finding indicates that HAND reported during this early stage may not be due to HIV alone, raising the question of whether simply controlling HIV replication during early infection will have a neurological benefit.

### Impact of Early Antiretroviral Treatment

Though results of recent studies of early infection challenge the prevailing assumption that HIV-associated neurologic injury occurs only in the late stages of infection, these data do not yet prove that CNS infection, inflammation, and neural injury during this early period are predictive of neurological outcomes. Furthermore, it is unclear whether the early initiation of antiretroviral therapy may protect the CNS. Immediate initiation of ART in subjects identified in the very early acute Thai cohort has been associated with improvement in inflammatory brain metabolites measured through proton magnetic resonance spectroscopy (MRS), with median levels reduced to those measured in HIV-uninfected control subjects [45]. However, in this study, individual subjects with the highest baseline inflammatory metabolite measures had persistently elevated levels of brain inflammation after treatment, suggesting a failure of complete cessation of CNS inflammation with therapy in some subjects despite extremely early initiation of ART. This finding is consistent with the more general observation that CSF immune activation, brain inflammation detected by MRS, and microglial activation in brain tissue persist in patients on long-term suppressive ART started during the chronic stage of infection [40, 58–61]. Persistent CNS immune activation associates with levels of detectable CSF HIV RNA in suppressive ART and brain HIV DNA in presymptomatic subjects [41, 59, 62], indicating that CNS inflammation may be a surrogate for magnitude or activity of brain HIV reservoirs. Further studies are needed to indicate whether very early initiation of ART may effectively abrogate HIV infection and inflammation within the CNS. Studies of neuropsychological performance in these subjects prior to and longitudinally after immediate initiation of ART are ongoing.

The overall concept that ART initiation prior to advanced immunosuppression may be beneficial to the CNS has been supported by observations that CD4 nadir is an important predictor of neurocognitive performance in treated subjects with chronic infection [2••, 63, 64]. A recent report examining HIV-infected and uninfected persons affiliated with the US military [65] has more directly assessed whether a short duration of infection prior to treatment initiation may be associated with preservation of neurologic and cognitive function. The 200 HIV-infected subjects enrolled in this study had prior routine testing, resulting in known seroconversion within less than a 1.5 year window. These subjects also had low prevalence of substance use and free access to HIV care from early in the disease course such that the majority had initiated ART within approximately 3 years of initial HIV infection. Rates of neurocognitive impairment based on neuropsychological testing were similar between these early-treated HIV infected subjects and 100 HIV-uninfected matched subjects, suggesting that a shortened duration of infection prior to treatment may be beneficial to the CNS.

## Emerging Issues Relating to the CNS in the Setting of ART

While classical HIV associated dementia (HAD) and progressive milder forms of disease may be presumed to be secondary to unchecked viral replication and inflammation within the CNS, HAND which persists or develops on ART is less well understood. The etiology of incident neurocognitive dysfunction in ART-suppressed patients is likely multifactorial, but may include poor access of ART to cells and tissues harboring HIV infection within the CNS, smoldering HIV infection which ‘escapes’ treatment and may be facilitated by immune activation, and potential antiretroviral toxicity in the nervous system.

### Antiretroviral Treatment Strategies for CNS Infection

Due to barriers separating the peripheral circulation and the brain and CSF compartments, levels of antiretroviral drugs are reduced in CNS tissues, leading to speculation that systemically effective ART might fail to control CNS HIV infection. For the most part, ART appears to exert salutary effects in suppression of CSF HIV RNA and inflammation even when it fails to achieve peripheral viral control [66–68]. Similarly, the dramatic effect of ART in reduction of incidence of HAD supports the overall success of ART within the CNS compartment [69]. However, more subtle forms of HAND may persist in part due to failure of typical ART regimens to completely halt HIV replication in the brain due to reduced CNS drug exposure. CSF HIV RNA is generally lower than that in plasma, and typically decays in parallel to plasma viral load in response to ART. However, in some cases, CSF decay is delayed in response to ART [66], which may be accounted for by poor CNS penetration, delayed attenuation of replication within long lived cells such as tissue macrophages, or locally compartmentalized HIV variants with distinct resistance profiles.

In recognition of the fact that due to steric or molecular properties, certain antiretrovirals have decreased potential access to the CNS, Letendre and colleagues developed a CNS penetration-effectiveness (CPE) score to quantitate the putative CNS effects of each drug, taking into account to varied degrees the relative concentrations of each drug in CNS tissues, its ability to reduce CSF load, or affect dementia. Within each class of antiretrovirals, a CPE value is assigned to each drug, with higher values associated with presumed higher CNS effectiveness, and a total CPE score for a regimen calculated by adding the individual drug scores together [70, 71]. While higher CPE regimens have been shown to better suppress CSF HIV RNA levels [71, 72], studies examining the relation of CPE with neurologic and cognitive performance have shown conflicting results. Although in a few longitudinal studies, subjects with HAND showed better improvement with initiation of higher CPE regimens, results of cross sectional studies have shown variable results, possibly in part due to initial selection of high-CPE regimens for more medically or neurologically ill patients [73–77]. Since macrophage and microglial cells are the primary cells within the CNS productively infected by HIV, recent investigation has focused on the role of antiretroviral penetration into monocytes, cells which may serve as circulating precursors to these cellular reservoirs within the CNS and which have been found by some investigators to be linked to presence of HAND [78, 79]. Putative ability of individual antiretrovirals to affect HIV replication within monocyte-lineage cells has been quantitated as a monocyte efficacy (ME) score, and one study employing summed ME scores from drug regimens found this to be a closer predictor of neuropsychological performance than CPE [80]. Ideally, randomized clinical trials of higher as compared to lower CPE and ME regimens would provide the best data to indicate whether such indices should be taken into account in treatment of HIV infected individuals and specifically those meeting criteria for HAND. Such studies are complex and have thus far proved challenging to implement but current studies are in progress or in development which should yield new insight into these issues.

### Progressive CNS Disease in Well-Treated Patients

For the vast majority of HIV-infected persons, systemically suppressive ART has resulted in apparent protection of the CNS from severe progressive neurologic disease, likely through a combination of reduced HIV replication within the CNS itself, reduced systemic as well as CNS immunoactivation, and reduced trafficking of immune cells as well as virally-infected cells to the nervous system. However, recent reports have drawn attention to the fact that in some rare cases, substantial HIV infection, inflammation, and clinical disease can evolve and manifest within the CNS in the setting of HIV treatment. In the most dramatic examples, patient can have relative immune preservation or reconstitution and undetectable plasma viral loads for prolonged periods, but still experience emergent CNS disorders. When explored carefully, the common features of patients with these conditions include stable ART therapy, well-controlled plasma viremia either to consistently undetectable levels or with low-level ‘blips,’ and new and progressive clinical signs and symptoms including new encephalopathy manifesting as dementia or aphasia, often accompanied by tremor, ataxia, severe headache, or sensory changes [81••, 82–86]. Upon CSF HIV RNA quantitation, these patients have findings of CSF HIV RNA levels one log_10_ greater than plasma levels or >50 copies/ml where plasma levels are <50 copies/ml. CNS inflammation manifesting as CSF pleocytosis or increased CSF protein or albumin ratio is also typically associated, features not present in typical patients on ART. While some of these reports have included patients with very low CD4 counts or on monotherapy or salvage therapy, others have described symptomatic CSF escape in persons with reconstituted immune systems on standard regimens. Because of the progressive neurological dysfunction noted in the context of CSF to plasma ‘discordance’ with a pattern different from that in usual untreated or treated HIV, this syndrome has been referred to as ‘symptomatic CSF escape.’ These syndromes with manifest clinical neurologic disease are distinct from ‘asymptomatic CSF escape’ which is detected in a proportion of HIV-infected individuals undergoing lumbar puncture without symptoms and which has unclear clinical significance [62].

Though the lack of suppression of CSF HIV RNA is a hallmark of the syndrome, the pathogenesis most likely involves not only the CSF and meninges, but the brain parenchyma itself, based on the clinical manifestations of the disorder, neuroimaging which has been reported in several cases to demonstrate patterns suggestive of cerebral inflammation, and brain pathology which indicates CD8 T lymphocyte infiltration and widespread inflammation. Indeed, these syndromes are likely on a continuum with newly described cases of ‘CD8 T cell encephalitis’ in which patients with incident and progressive dementia or clinical encephalitis have been noted to have dramatic infiltration of CD8 T lymphocytes on biopsy with imaging and CSF evidence of inflammation as well as low-level detectable CSF HIV [87•, 88]. Though these syndromes are primarily described as encephalitis from a pathological standpoint, the clinical spectrum of these conditions likely overlaps with those described primarily based on CSF analysis as CSF escape cases (see Table 1). Based on the high CD4 T cell counts of subjects manifesting encephalitis as well as the fact that the dramatic inflammatory changes detected pathologically are not accompanied by high levels of HIV or other antigens, these disorders have been considered a variant of immune reconstitution inflammatory syndrome (IRIS) and thus treated in some cases with immunosuppressants. Initiation of ART has been previously identified to be associated with emergence of leukoencephalopathy and inflammation suggestive of an IRIS syndrome related directly to HIV in the brain [89]. In these recent reports of CD8 encephalitis as well as CSF escape, though inflammation similarly appears central to the pathology, many cases seem to arise in the context of stable therapy, suggesting a complex, perhaps chronic form of IRIS.

**Table 1:** Recent series of acute or subacute clinical syndromes of symptomatic HIV CSF escape or CD8 encephalitis

First author, year	Sample size	CD4 median, range	CSF HIV RNA level median, range	Clinical correlates	CSF WBC median, range	Other correlates	Treatment
Canestri 2010 [81••]	11	432 cells/mm^3^ (range, 107–631 cells/mm^3^)	880 copies/mL (range, 558-12,885 copies/m)	Cognitive impairment, headache, ataxia	31 cells/ μl (range 6–270)	5/8 with genotyping had CSF resistance to current regimen	Adjusted regimen for CSF resistance and CNS penetration
						2 receiving monotherapy at onset	
Peluso, 2012 [53•]	10	482 cells/mul (range 290–660)	3900 copies/ml (range 134–9056)	Cognitive impairment, ataxia, tremor	14.5 cells/μl (range 0–200)	6/7 with CSF genotyping had CSF resistance to current regimen	Adjusted regimen for CSF resistance and CNS penetration
Lescure, 2013 [87•]	14	212/μL (range, 84–742/μL)	5949 copies/mL (range, 0–36242 copies/mL)	Cognitive impairment, headache, seizures	35 cells/μl (range 1–220)	2 off of ART	Corticosteroids

CSF = cerebrospinal fluid; WBC = white blood cell count

Both symptomatic CSF escape and CD8 encephalitis are likely related to ongoing CNS infection in the CNS compartment in the context of a substrate of CNS inflammation, usually in the context of a relatively reconstituted immune system. In many described cases, genotyping of CSF yields a distinct resistance pattern in the CNS compartment from that present in plasma, or evidence of resistance to drugs which successfully suppress plasma virus. This suggests that one contributor to persistent HIV production in these disorders may be compartmentalization of HIV strains within the CNS, perhaps facilitated by poor CNS exposure of ART or by prior inconsistent medication adherence. Additionally, the presence of elevated CNS inflammation in symptomatic CSF escape and CD8 encephalitis may directly lead to enhanced production of HIV locally within CNS tissues, as inflammatory signals lead to increased trafficking of infected cells, as well as target cells of infection, to the CNS. Many patients with these disorders have been reported to have clinical improvement when ART regimens are tailored both to address resistance genotypes detected in the CSF as well as to improve presumed blood brain barrier penetration. For this reason, knowledge of these syndromes is critical to the HIV provider and patient, since substantial clinical improvement or even complete resolution of symptoms may result from proper diagnosis and treatment intervention. Additionally, recognition that such conditions can exist provides proof for the concept that a biologically meaningful reservoir for HIV infection may exist in the CNS even in the setting of treatment. In one report, CSF escape was accompanied by recrudescence of low-level plasma viremia, which was hypothesized to possibly have resulted from ‘reseeding’ of the successfully treated periphery from the CNS compartment [84]. A number of independent studies have suggested that inflammation and brain injury may persist in the CNS in individuals on ART [58, 60, 90]. Overt clinical syndromes may be the most dramatic examples of a chronic low level activity within the CNS despite standard ART.

### Potential Neurotoxicity of ART

A substantial controversy in the field of neuroAIDS is whether antiretroviral therapy may be directly toxic to the nervous system and itself contribute to HAND. Though numerous studies and epidemiological observations have demonstrated the major beneficial effects of effective ART for the CNS, one study demonstrated improvement in neuropsychological testing performance in a cohort of 167 HIV infected persons interrupting therapy [91]. Though some improvement may be observed in individual performance on repeated testing, the study investigators felt that the improvement observed in this case was more than would be expected from a practice effect. Numerous medications in current use have been postulated to have neurotoxic potential in the CNS. However, the mechanisms of these effects are unknown, and the limited studies supporting them are based on *in vitro* models that have unclear clinico-pathologic significance [92, 93].

Individual agents have potential or observed adverse effects on the CNS. The most dramatic example of these is the NNRTI efavirenz, which has a broad array of cognitive and behavioral effects including insomnia and vivid dreaming, daytime encephalopathy, fatigue, hallucinations, irritability, increased anxiety and suicidality. Though most of these effects are at their peak in the first weeks after initiating therapy, many persist long term and are a central reason for discontinuing or switching efavirenz-containing regimens [94]. Not only do patients experience symptoms of disturbed sleep, cognition and mood, but cognitive impairment may associate with use of efavirenz. In a recent study of 146 asymptomatic subjects assessed in routine follow-up visits to an outpatient clinic, treatment with an efavirenz-containing regimen was independently associated with impairment on neuropsychological testing and poor performance on specific tests [95]. Since these subjects did not have symptoms of impairment, selection bias for simpler regimens in patients with existing deficits is unlikely to have confounded this finding. Similarly, in subjects interrupting ART, those on efavirenz containing regimens had greater improvement in neuropsychological performance than those on regimens without efavirenz [91]. A broad antiretroviral neurotoxicology study identified efavirenz as one among several antiretrovirals which damaged neurons when directly applied in vitro [93]. Recently, investigators more specifically identified damage to neuronal dendrites by metabolites of efavirenz at concentrations readily detected in CSF of patients receiving efavirenz containing regimens [92]. However, a long-term study of subjects followed for 3 years on efavirenz containing regimens showed stability of neuropsychological testing performance in subjects with normal testing at baseline, suggesting if tolerated, efavirenz does not appear to lead to accumulated neurotoxicity which is clinically detectable [94]. Importantly, the ease of consistently adhering to efavirenz-containing regimens in terms of convenience of dosing must be weighed against potential neurotoxicity in terms of cost-benefit to the CNS. Overall, the benefit conferred by ART in protecting the CNS likely outweighs any chronic neurotoxic effects, but in the case of efavirenz, acute psychologic effects may be a rationale for switching drugs. Further prospective, longitudinal studies are warranted to assess the potential deleterious effects of various antiretroviral regimens on the CNS.

## Conclusion

Despite the widespread use of ART and its proven effectiveness in preventing classical HIV-associated dementia, a substantial proportion of HIV infected individuals meet criteria for HAND [2••, 7, 8]. Addressing this persistence of neurologic and cognitive symptoms in HIV infected persons is a major challenge to optimizing quality of life in persons living with HIV. One possible explanation is that a CNS HIV reservoir for infection and inflammation is established in the early stages of infection, before HIV is typically diagnosed, and this substrate for HIV-related brain injury leads to irreversible damage within the CNS before ART is initiated. Another potential explanation is that in the setting of apparently systemically suppressive ART, processes of infection, inflammation, and brain injury may be attenuated but remain active, allowing for progressive impairment on treatment. Finally, other sequelae of treated HIV infection may contribute to cognitive impairment, even if classical HIV encephalitis is eliminated with ART. Future efforts should focus on earlier diagnosis and treatment of HIV as well as on identification of optimal antiretroviral and adjunctive therapies to reduce residual infection and neurotoxicity within the CNS.
